# Microwave-Assisted Surface Modification of Metallocene Polyethylene for Improving Blood Compatibility

**DOI:** 10.1155/2013/253473

**Published:** 2013-06-12

**Authors:** Hemanth Mohandas, Gunalan Sivakumar, Palaniappan Kasi, Saravana Kumar Jaganathan, Eko Supriyanto

**Affiliations:** ^1^Department of Research and Development, PSNA College of Engineering and Technology, Kothandaraman Nagar, Dindigul, Tamil Nadu 624 622, India; ^2^Cardiovascular Engineering Centre, Faculty of Bioscience and Medical Engineering, Universiti Technologi Malaysia, 81310, Johor Bahru, Johor, Malaysia

## Abstract

A wide number of polymers are being used for various medical applications. In this work, microwave-assisted surface modification of metallocene polyethylene (mPE) was studied. FTIR analysis showed no significant changes in the chemical groups after treatment. Contact angle analysis revealed a decrease in contact angle of the treated samples insinuating increasing hydrophilicity and better biocompatibility. Qualitative analysis of treated samples using scanning electron microscope (SEM) depicted increasing surface roughness and holes formation further corroborating the results. Coagulation assays performed for estimating prothrombin time (PT) and activated partial thromboplastin time (APTT) showed an increase in the clotting time which further confirmed the improved blood compatibility of the microwave-treated surfaces. Further, the extent of hemolysis in the treated sample was lower than the untreated one. Hence, microwave-assisted surface modification of mPE resulted in enhanced blood compatibility. Improved blood compatibility of mPE may be exploited for fabrication of artificial vascular prostheses, implants, and various blood contacting devices.

## 1. Introduction

One of the most versatile classes of biomaterials is polymers. It has been widely used in medicine and biotechnology as well as in the food and cosmetics industries [1]. Polymer applications include surgical devices, implants, and supporting materials (e.g., artificial organs, prostheses, and sutures), drug-delivery systems, carriers of immobilized enzymes and cells, biosensors, components of diagnostic assays, bio-adhesives, ocular devices, and materials for orthopedic applications [2]. Most of the polymers have the desired bulk physical and mechanical properties to be used as implants but many of them do not have the desired blood compatibility. The best method to enhance the blood compatibility of the polymer is to undergo proper surface treatments on the polymer.

Recent developments in metallocene single-site catalyst technology produced a new class of polyolefins having improved performance properties like enhanced toughness, sealability, clarity, and elasticity [3]. Metallocene polyethylene (mPE) is one among these polyolefins processed using metallocene. Metallocene consists of two cyclopentadienyl anions (Cp, C_5_H_5_^−^) bound to a metal center (M) with the oxidation state II, resulting in a general formula (C_5_H_5_)_2_ M [3, 4]. These materials have a high potential as replacements for flexible PVC in the coming years as their film density is approximately 30% lower than that of PVC, creating a lower volume of waste material from disposable medical devices [5]. Current medical applications of mPE include disposable bags, storage bottles, blood bags, and syringe tubes. mPE has an excellent permeability to oxygen and acts as a barrier towards ammonia and water makes mPE a plausible candidate for blood contacting devices and medical implants.

One of the major concerns of these devices is the lack of blood compatibility of their surfaces. Some examples are the occlusion of small-diameter vascular grafts and failure of blood-contacting biosensors due to thrombus formation on the device surface [6]. Other instances include embolism and thrombocytopenia (platelet consumption) produced by the blood-contacting biomedical devices. Hence, anticoagulant drugs have to be administered for long-time or even for their life term [7]. Furthermore, side effect like the chance of hemorrhage will be greatly increased.

The polymer surface properties are mostly controlled by the chemical nature and morphology of their own surfaces [8, 9]. The surface modification processes should not distort the bulk or the mechanical properties of the polymers. A number of methods have been introduced in the literature which helps in the surface modification of the polymeric material. Some of the surface treatments are: chemical and mechanical methods, grafting co-polymerization [10], plasma treatment [11], UV and laser irradiation [12], dielectric discharge [13], and microwave plasma irradiation [14]. The above-said methods can be grouped into two main categories, namely, the methods which modify the morphology and chemical properties of the polymer and the methods that produce a thin layer of different substance on the surface of the polymer. 

Microwaves are radio waves with wavelengths ranging from as long as one meter to as short as one millimeter, or equivalently, with frequencies between 300 MHz (0.3 GHz) and 300 GHz. Microwaves are used widely in medical applications like computed tomography (CT), microwave ablation, surgery, and so forth. Recently microwaves have been used successfully for surface modification of polymers and fabrics [15–17]. The cost, time, and energy needs of microwave treatment are considerably lower than other modes. Moreover, the size of the microwave system is compact while compared with other methods because of the high energy applicators and direct energy absorption by most of the materials. Further, a good instantaneous control and reduced environment pollution are some of the key benefits which promote microwave treatment as a better tool for surface modification [18]. In this investigation, mPE film is exposed to microwaves, and its changes in the surface properties and blood compatibility were evaluated.

## 2. Materials and Methods

### 2.1. Sample Preparation

mPE was cut into samples of square shape with a length and breadth of 1 cm × 1 cm for the studies. The samples were washed twice with distilled water (for 2 minutes) to clear its surface and wiped with 70% ethanol to make free from foreign particles before exposure to microwaves. All experiments were performed on the same day of the treatment.

### 2.2. Microwave Treatment

The microwave radiation was produced with the help of microwave oven Onida power grill 17 DL producing a frequency of 2450 MHz with 1200 W as rated microwave input. The cut samples were treated in the microwave oven for the specified time intervals before being analyzed for its surface changes and blood compatibility.

### 2.3. Surface Characterization Tests

To observe the changes associated on the surface of the untreated (control) and treated samples following characterization techniques were employed. These experiments were aimed to observe the chemical changes, wettability, and microstructure of the samples before and after treatment.

#### 2.3.1. Attenuated Total Reflectance Fourier Transform Infrared Spectroscopy (ATR-FTIR)

The chemical changes in the polymer after treatment were investigated by Attenuated Total Reflectance Fourier Transform Infrared Spectroscopy (ATR-FTIR). ATR-FTIR spectra was recorded with Fourier Transform Infrared Spectrometer Model FTIR-6300 (Japan-made) equipped with DLATGS detector. Diamond is used as the ATR crystal. Three samples, namely, untreated and 5- and 15-minute treated were taken for ATR-FTIR analysis.

#### 2.3.2. Contact Angle Measurements

Measurement of contact angle formed between the water drop and the surface of the sample can provide us the evaluation of wettability of the samples. The measurement was done by using Dynamic Contact Angle Analyzer (FTA 200—First Ten Angstroms). This action appears live on the computer screen and the salient images are captured to the computer's memory for later image analysis. A needle of 0.55 mm in diameter (Dispovan) was used to place a drop of water onto the sample surface. A photograph was taken after placing the water droplet on the sample for 30 seconds and the contact angle was measured with the help of computer-interfaced program.

#### 2.3.3. Scanning Electron Microscope (SEM)

To observe the surface morphology changes associated with microwave treatment, scanning electron microscopy (JEOL, JSM-638OLA) was employed. Samples were subjected to double sputtering with gold before obtaining images from SEM. The SEM images were obtained at a magnification of 1000x and 2500x.

### 2.4. Coagulation and Hemolysis Assays

Coagulation assays were used to measure the polymer-induced abnormalities in the blood clotting cascade. End-point of these assays was the onset of fibrin clot when the platelet-poor plasma comes in contact with the control and treated microwave sample surfaces. Hemolysis assay were performed to estimate the damage to the red-blood cells.

#### 2.4.1. Prothrombin Test

Measurement of prothrombin time serves as a yardstick to assess the interdiction of the extrinsic pathway. Platelet poor plasma (PPP) (100 mL at 37°C) was applied on the surface of the control and treated substrates along with NaCl-thromboplastin (Factor III; 100 mL Sigma) containing Ca^2+^ ions. The time taken for the onset of fibrin clot was estimated using stopwatch and a steel hook (*n* = 3) [19].

#### 2.4.2. Activated Partial Thromoplastin Test (APTT)

APTT is widely used to assess the ability of the blood to coagulate through the intrinsic pathway and to assess the effect of biomaterial on possible delay of the process. Surfaces of the samples were preincubated with PPP (100 mL at 37°C) before the addition of rabbit brain encephalin. These mixtures were incubated for 5 min and then calcium chloride (0.025 M) was added to the mixture. The time for initiation of clot formation, detected by using a steel hook, was measured using a stopwatch (*n* = 3) [19].

#### 2.4.3. Hemolysis Test

Both untreated and treated sample (15 min) were equilibrated with physiologic saline (0.9% w/v; 37°C, 30 min) and then incubated with 3 mL aliquots of citrated blood diluted with saline (4 : 5 ratios by volume). The positive control taken was the mixture of blood and distilled water in the ratio 4 : 5 by volume to cause complete hemolysis. The negative control is the physiological saline solution which produces no coloration. The samples were incubated in their respective mixtures (60 min, 37°C). These mixtures were then centrifuged and the absorbance of the clear supernatant was measured at 542 nm. The absorbance of positive control was normalized to 100% and the absorbance of different samples was expressed as a percentage of hemolysis compared with their positive control [19].

## 3. Results

### 3.1. Attenuated Total Reflectance-Fourier Transform Infrared Spectroscopy (ATR-FTIR)

**Figure 1:**
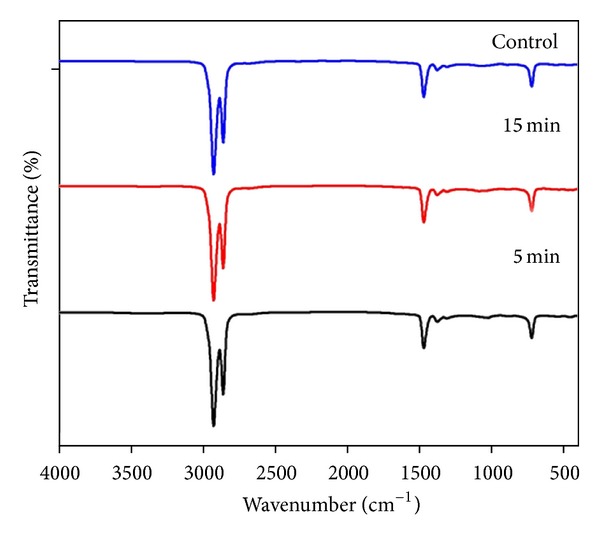
FTIR spectra of control and microwave-treated metallocene polyethylene.

Attenuated Total Reflectance-Fourier Transform Infrared Spectroscopy (ATR-FTIR) was done to explain the chemical changes of the polymer before and after the microwave treatment.  Figure 1 shows the results obtained from the ATR-FTIR. Comparing the treated (exposure times: 5 min and 15 min) and untreated samples, there were no dramatic changes observed in adding or losing any groups of the polymer. Hence, microwave treatment does not significantly influence the chemical structure of the polymer. Initial two peaks observed at 2850 cm^−1^ and 2930 cm^−1^ were found out to be alkane group (C–H stretch). The next peak absorbed between 1470 and 1450 cm^−1^ and the last small peak between 725 and 720 cm^−1^ also belong to the alkane's family with differences in its structure. Peak at the range of 1470–1450 cm^−1^ may be due to C–H bend and the last peak at the range 725–720 cm^−1^ is due to C–H rock. 

### 3.2. Contact Angle Measurement

**Figure 2:**
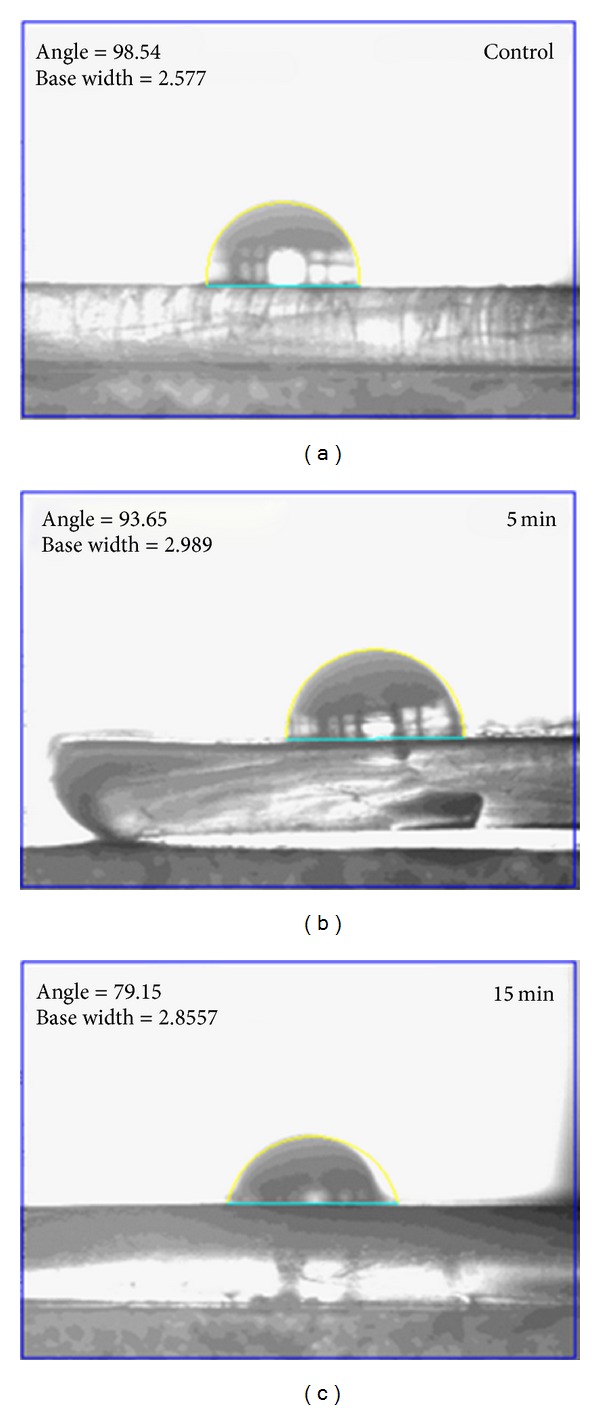
Water contact angle of control and microwave-treated metallocene polyethylene.

Contact angle measurements were performed on both treated (exposure times: 5 min and 15 min) and control sample to explain the wettability or the hydrophilicity of the samples. Results of the contact angle measurements clearly indicated that the value of contact angle of treated samples is lower than the control (Figure 2). Control sample has a contact angle of 98.54°, whereas the treated samples found to possess an angle of 93.65° (5 min sample) and 79.15° (15 min sample). The value of contact angle decreased to a much lower value for higher exposure time. Thus, the decrease in contact angle signifies increase in the wettability and hydrophilicity of the treated samples.

### 3.3. Scanning Electron Microscope (SEM)

**Figure 3:**
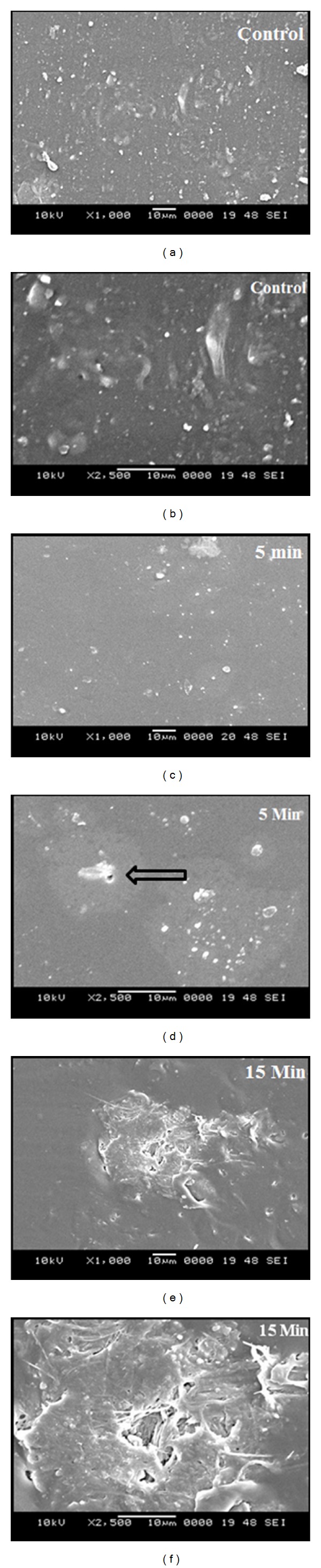
SEM micrographs of control and microwave-treated metallocene polyethylene. Arrow mark indicates the pitted surface.

Scanning electron microscope images were taken to visualize the changes induced in the surface of the microwave-treated samples. To perform this analysis, as in the case of contact angle measurements, untreated sample and 5 min and 15 min treated samples were utilized for this study. The SEM images provided a qualitative type of analysis by visualizing the change in the surface of the polymer. SEM images of the 5 min treated sample displayed minor surface changes in their morphology compared to the control. But, on prolonged treatment of the sample for 15 min, microwave-induced significant surface changes are evident. There was an increase in the surface roughness, and also pitted surface formations were seen at certain regions of treated samples (Figure 3).

### 3.4. Coagulation and Hemolysis Assays

**Figure 4:**
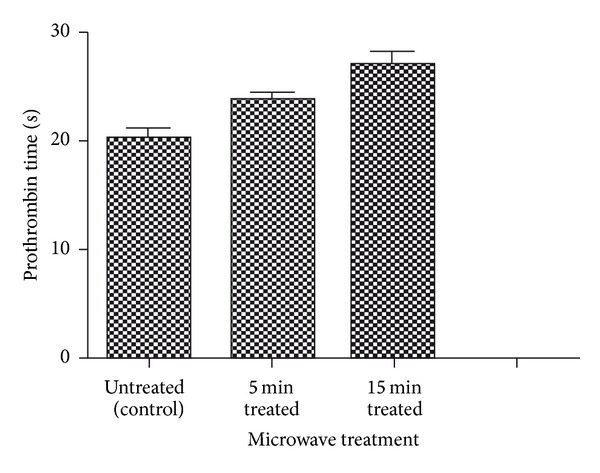
Comparison of prothrombin time (PT) of control and microwave-treated metallocene polyethylene (*n* = 3).

**Figure 5:**
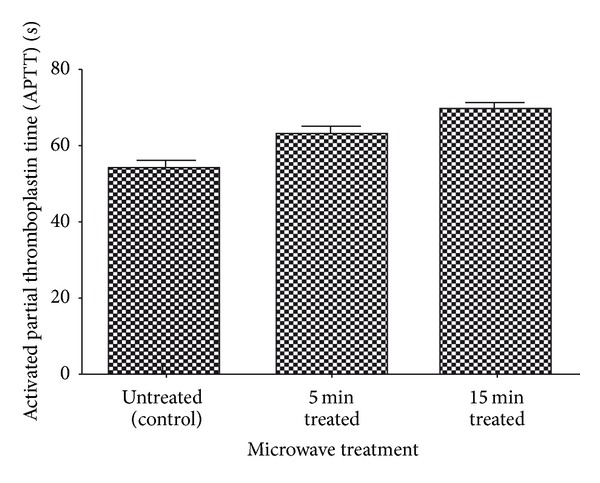
Comparison of activated partial thromboplastin time (APTT) of control and microwave-treated metallocene polyethylene (*n* = 3).

**Figure 6:**
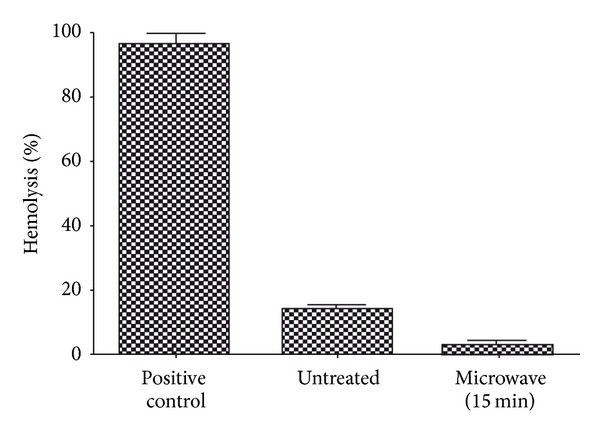
Percentage hemolysis of control and microwave-treated metallocene polyethylene (*n* = 3).

Blood coagulation assays of the above-mentioned three samples (control, 5 min and 15 min microwave exposed) were performed. Both PT and APTT time of treated samples were found to be increased when compared with the untreated control sample (Figures 4 and 5). Statistical analysis of the control sample with the treated ones using one-way ANOVA indicated significant differences (*P* < 0.05) between them for both PT and APTT times. Results of hemolysis assay indicated that untreated sample induced 14.38% hemolysis, whereas the 15 min microwave exposed sample showed only 3.26% after 60 minutes exposure to blood (Figure 6). In summary, microwave treatment of mPE had increased the time for coagulation and also reduced the hemolysis ratio significantly.

## 4. Discussion

Polymers have gained a widespread attention in the field of biomaterials especially for fabrication of implant materials. mPE is an emerging polymer that has been used in manufacturing of blood bags, bottles, packaging materials, syringe tubes, and so forth due to its excellent strength and optical properties. However, when mPE is considered for blood contacting devices, lack of blood compatibility is a major concern. To improve the blood compatibility, various surface modifying techniques are employed. Microwave-assisted surface modification of mPE and its potential for improving blood compatibility were studied.

FTIR results of the untreated and treated samples indicated that there is no difference in the functional groups associated with microwave treatment [15–17]. The results obtained were also confirming with the same previously concluded researches which could not find any significant difference in the chemical groups after microwave treatment. We could not observe the broad absorption peak at 3400–3500 cm^−1^ (hydroxyl groups) as observed with the case of microwave oxygen plasma treated PDMS surface [17]. This may be due to the power and duration of treatment of the microwave oven (700 W for 5 and 15 min) which was higher compared with them (180 W, 4 min). But the results obtained are greatly confirming with the microwave treatment of wool at 400 W for 3 min, where they could not find any absorption peak at 3400–3500 cm^−1^ corresponding to the hydroxyl groups [15].

Contact angle measurement indicated decreasing angles for the treated polymers compared with control. A decrease in the contact angle indicates that the surface is with higher degree of wettability and hence improved biocompatibility [20]. Decreasing contact angle may be attributed to the chemical or morphological changes associated with the polymer [21]. Since, the FTIR studies of the samples did not reveal any chemical changes associated with microwave treatment, the increase in the wettability or hydrophilicity may be better explained from the morphological studies conducted on the polymer. SEM images indicated increasing surface roughness on the treated polymers. There was also pitted surface formation in the microwave exposed surface of the polymer, which elucidates the better wettability of these treated polymers. To culminate, increasing surface roughness and associated decrease in the contact angle were in accordance with the several researches which illustrated increased wettability of polymers [20–22].

Increasing hydrophilicity and wettability is linked with the better biocompatibility of the polymer [20]. The results from the coagulation assays showed increase in the PT and APTT time of the microwave-treated polymers. The extent of hemolysis in the treated samples was lower than the untreated one. This further corroborates that microwave treatment enhances the blood compatibility of the mPE. These results were in agreement with some recently published works [19, 23]. Hence microwave-assisted surface modification might be a probable strategy to improve the blood compatibility of the mPE. Improved blood compatibility of mPE may be exploited for fabrication of artificial vascular prostheses, implants, and various blood contacting devices. Further, platelet adhesion assay and some *in vitro* cell culture of fibroblast cells on the surface of treated polymers will further glorify the results obtained and promote mPE as a plausible candidate for biomedical applications.

In summary, microwave-assisted surface modification of mPE was investigated. FTIR analysis showed no significant changes in the chemical groups after treatment. Contact angle analysis revealed a decrease in contact angle of the treated samples insinuating increasing hydrophilicity and better blood compatibility. Qualitative analysis of treated samples using scanning electron microscope depicted increasing surface roughness and holes formation further corroborating the results. Coagulation assays performed for estimating prothrombin time (PT) and activated partial thromboplastin time (APTT) showed an increase in the clotting time which further confirmed the improved blood compatibility of the microwave-treated surfaces. Improved blood compatibility of mPE may be exploited for fabrication of artificial vascular prostheses, implants, and various blood contacting devices.
